# Resources That Improve Medical Board Licensing Examination Performance

**DOI:** 10.7759/cureus.5927

**Published:** 2019-10-16

**Authors:** Frank Jackson, Ethan Duane, Robert Harmon, Ryan A Kollar, Nicole M Rainville, Ryan M Smith

**Affiliations:** 1 Obstetrics and Gynecology, Saint Francis Hospital and Medical Center, Hartford, USA; 2 Anesthesiology, College of Osteopathic Medicine, University of New England, Biddeford, USA; 3 Miscellaneous, College of Osteopathic Medicine, University of New England, Biddeford, USA; 4 Obstetrics and Gynecology, College of Osteopathic Medicine, University of New England, Biddeford, USA; 5 Psychiatry, College of Osteopathic Medicine, University of New England, Biddeford, USA

**Keywords:** usmle step 1, comlex-usa level 1, board examination, medical school

## Abstract

Purpose

Examine the factors improving performance on national medical licensing board examinations.

Rationale

Accreditation Council for Graduate Medical Education (ACGME) accredited residency programs report the United States Medical Licensing Examination (USMLE) Step 1 and Comprehensive Osteopathic Licensing Examination-USA (COMLEX-USA) Level 1 scores as the most important criteria in selecting candidates to interview.

Hypotheses

(1) Certain resources are superior for exam preparation.

(2) Certain practice tests better assess exam preparedness.

(3) USMLE performance will correlate with the COMLEX-USA.

Methods

One-hundred and two (102) medical students were surveyed regarding preparation for and performance on COMLEX-USA Level 1 and USMLE Step 1.

Results

USMLE-specific question banks were positively correlated with performance on COMLEX-USA Level 1 and USMLE Step 1 while COMLEX-specific question banks showed no correlation. National Board of Medical Examiners (NBME) Comprehensive Basic Science Self Assessment (CBSSA) and National Board of Osteopathic Medical Examiners (NBOME) Comprehensive Osteopathic Medical Self-Assessment Examination (COMSAE) practice examinations were positively correlated with performance on the USMLE Step 1 and the COMLEX-USA Level 1. Scores on USMLE Step 1 and COMLEX-USA Level 1 were highly correlated. Students who took USMLE Step 1 performed better on COMLEX-USA Level 1 than those who did not.

Conclusion

COMLEX-specific resources may not adequately prepare students for COMLEX-USA Level 1. Students studying for COMLEX-USA Level 1 may benefit by preparing for USMLE Step 1.

## Introduction

Each year, both the number of U.S. medical schools and their respective class sizes continue to grow, dramatically outpacing the expansion and related availability of postgraduate year (PGY)-1 medical residency positions [1]. Since 1993, there have been fewer available positions in American Council of Graduate Medical Education (ACGME) accredited post-graduate training programs than there were applicants seeking to match [2]. In the 2017 National Residency Matching Program (NRMP) match, a total of 35,969 applicants competed for only 28,849 PGY-1 positions, representing a ratio of applicants to positions of 0.8 [2]. Despite the upcoming merger of ACGME and American Osteopathic Association (AOA) accredited post-graduate training positions by 2020, the move would only increase the number of accredited ACGME PGY-1 positions by 3,109, assuming that all AOA programs participating in the 2017 National Matching Service (NMS) match were incorporated into a joint match [3].

This trend of continued growth in the domestic medical graduate pool has served as a catalyst for the increasing competition that all applicants face in attempting to match into a residency program. The literature is replete with surveys and analyses of criteria that are predictive of success in both interviewing and matching at prospective residency programs. Consistent throughout much of this material is the conclusion that performance on the first component of national medical licensing board examinations - either United States Medical Licensing Examination (USMLE) Step 1, published by the National Board of Medical Examiners (NBME), for US-MD students, international medical graduates, and osteopathic medical students, or the Comprehensive Osteopathic Medical Licensing Examination of the United States (COMLEX-USA) Level 1, published by the National Board of Osteopathic Medical Examiners (NBOME) for osteopathic medical students - is a standardized comparison tool that proves vital in both the screening and ranking of candidates for residency positions in the United States [4-5]. Additional incentives for bolstering student performance on standardized board examinations result from the importance that residency directors place on passing these examinations in a single attempt [4], the accreditation requirement that allopathic medical schools use these rates in program quality improvement [6], and the accreditation requirement of the Commission on Osteopathic College Accreditation (COCA) that osteopathic medical schools publicly report these rates [7].

In consideration of this necessity to excel in medical licensing board examinations, medical students are inclined to be rigorous, persistent, and strategic in their approach to preparation. Recent research in the fields of learning and cognition has generated evidence to better inform students and educators about the methodology most likely to lead to success in taking high-stakes examinations. In particular, a great deal of work has been conducted on assessing the use of retrieval practice (i.e.*,* formative testing) as a more efficacious manner in which to encode long-term memory when compared to elaborative processes (often consisting of passive repetition). While traditional schools of thought have presupposed that the retrieval of information such as that completed during testing scenarios only served to assess previously acquired knowledge, recent work suggests that retrieval practice can serve as a mnemonic enhancer and generate greater long-term retention than would elaboration [8-9]. Further, studies suggest that retrieval practice may promote knowledge acquisition that can be applied in novel contexts [9]. Retrieval practice may also contribute to a phenomenon known as the “forward effect,” a process through which new information presented after conducting retrieval practice is more effectively consolidated into long-term memory [10].

The aim of the present study was to examine several factors in national medical licensing board examination preparation that have the potential to directly influence performance outcomes. We sought to evaluate the association between the use of retrieval practice examination preparation methods (i.e.*,* the use of question banks) and performance on licensing examinations, in addition to evaluating retrieval practice efficacy amongst various commercial preparation products that were marketed to target preparation for either the USMLE or the COMLEX-USA. Finally, we explored the independent correlation between performance on the USMLE and COMLEX-USA examinations.

## Materials and methods

In 2017, the authors solicited survey participation from the 171 third-year osteopathic medical students then enrolled in the Class of 2019 at the University of New England College of Osteopathic Medicine. Of those solicited, 102 (60%) students elected to complete the authors’ survey. This population of students both prepared and sat for the COMLEX-USA Level 1 while 58 also both prepared and sat for the USMLE Step 1 during the 2016-2017 academic year. Survey participation was solicited via e-mail, with voluntary participation. The survey was open during the months of July and August of 2017. Responses were de-identified and no additional demographic information was collected in order to maintain the anonymity of the participants.

The survey was composed of five sections. Section 1 of the survey sought to elucidate the month the student began preparation, question banks used, question mode (tutor, timed, or both tutor and timed), question organization (randomized or organ system-specific), number of questions completed in each question bank, and number of questions repeated in each question bank. Section 2 was composed of questions pertaining to the tools used for study plan organization, flashcard use (complete or partial), video resource use (complete or partial), and book use (complete or partial). Section 3 consisted of questions about Comprehensive Osteopathic Self-Assessment Examinations (COMSAE) Forms A-E (date taken and corresponding score) and COMLEX-USA Level 1 (date taken, goal score, and actual score). The final question of this section inquired as to whether or not the student prepared for USMLE Step 1. If this question was answered in the affirmative, participants were directed to Section 4 while those participants answering in the negative were directed to Section 5. Section 4 was composed of questions regarding NBME Comprehensive Basic Science Self-Assessments (CBSSA) 12-13 and 15-19 (date taken and score) and USMLE Step 1 (date taken, goal score, and actual score). Section 5 consisted of inquiries into residency specialty interest and how well the college curriculum prepared students for COMLEX-USA Level 1 (Likert scale of 1-7, with 1 being “very poorly” and 7 being “very well”).

Linear regression was used to evaluate the relationships of continuous variables (USMLE Step 1 and COMLEX-USA Level 1 performance, the number of questions completed in the various question banks, and performance on practice exams). Survey responses were also analyzed using unpaired t-tests comparing parametric variables. Statistical analysis was performed using the R Statistical Programming Package [11]. This study was reviewed by the university institutional review board (IRB) and its protocol was determined to be exempt from further IRB review and oversight.

## Results

USMLE and COMLEX-USA relationship

**Figure 1:**
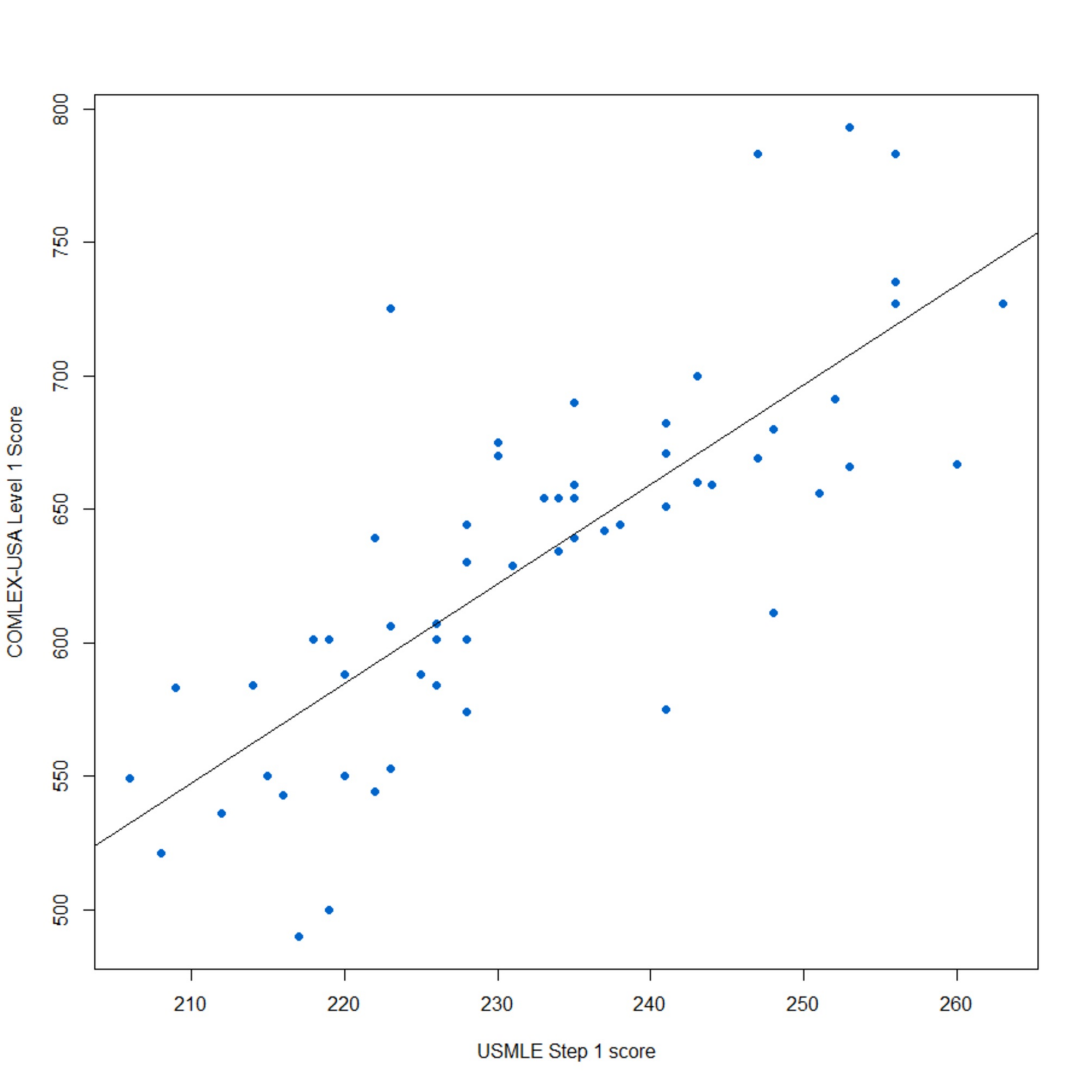
Relationship of USMLE Step I and COMLEX-USA Level 1 Scores USMLE: United States Medical Licensing Examination; COMLEX-USA: Comprehensive Osteopathic Medical Licensing Examination of the United States

**Figure 2:**
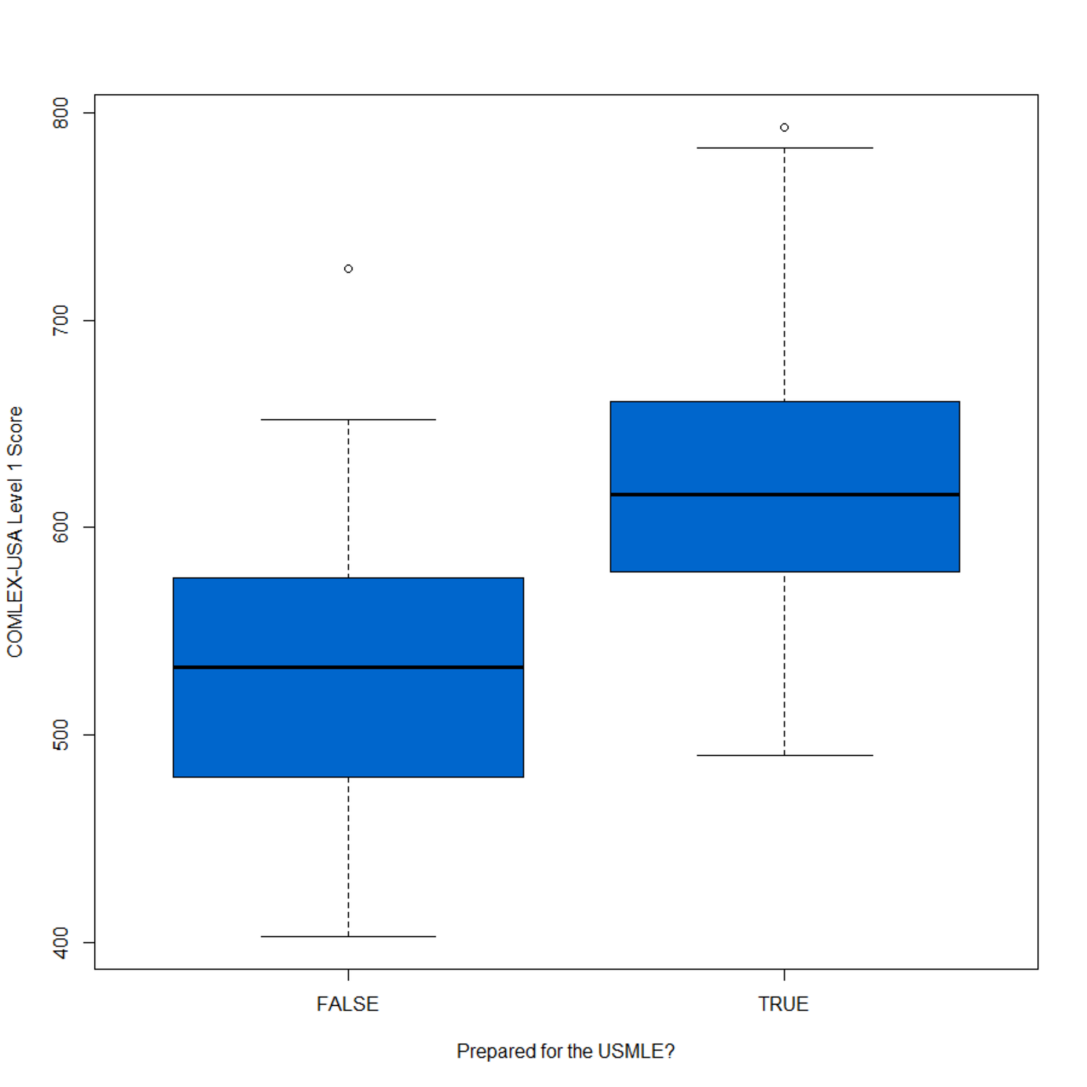
Boxplot of COMLEX-USA Level 1 Score based on preparation for USMLE Step 1 Exam status USMLE: United States Medical Licensing Examination; COMLEX-USA: Comprehensive Osteopathic Medical Licensing Examination of the United States

Fifty-eight students (57% of participants) reported taking both USMLE Step 1 and the COMLEX-USA Level 1. Performance outcomes for these examinations were strongly correlated (r = 0.78) and can be found in Figure 1, and students who reported taking the USMLE Step 1 performed better than those who did not, with those taking USMLE with a mean of 633 on the COMLEX-USA while students who did not have a mean of 553 (t=6.64, p < 0.001), see Figure 2.

Question banks

The total number of questions that students completed was positively correlated with performance on the USMLE Step 1 (r = 0.30) and COMLEX-USA Level 1 (r = 0.29). The number of questions answered in banks marketed for preparation for the USMLE, such as UWorld, USMLE Rx, and Kaplan USMLE, was positively correlated with performance on both USMLE Step 1 (r = 0.38) and COMLEX-USA Level 1 (r = 0.42). The number of questions answered in banks marketed toward preparation for the COMLEX, such as Truelearn COMBANK (question bank for COMLEX-USA), COMQUEST (question bank for COMLEX-USA), and Kaplan COMLEX, was poorly related to performance on the USMLE Step 1 (r = -0.03) and negatively correlated with performance on COMLEX-USA Level 1 (r = -0.22) (Table 1).

**Table 1:** Correlation of questions completed with exam performance USMLE: United States Medical Licensing Examination; COMLEX-USA: Comprehensive Osteopathic Medical Licensing Examination of the United States; COMQUEST and COMBANK (question banks for COMLEX-USA)

	USMLE Step 1 Performance	COMLEX-USA Level 1 Performance
Total unique questions	0.2954854	0.2855076
USMLE specific question banks	0.3824327	0.4222505
Unique UWorld questions	0.1846993	0.4366046
Unique USMLE Rx questions	0.3975825	0.3205712
Unique Kaplan USMLE questions	0.2145561	0.1336113
COMLEX-USA specific question banks	-0.02833969	-0.2168849
Unique Truelearn COMBANK questions	-0.1001551	-0.06868627
Unique COMQUEST questions	-0.004152736	-0.2323102
Unique Kaplan COMLEX questions	0.1361367	-0.09639769

Students who completed UWorld in timed mode performed better on COMLEX-USA Level 1 than those completing questions in tutor mode (t = 2.04, df = 34.67, p = 0.049). Students who completed Truelearn’s COMBANK in timed mode did better on COMLEX-USA Level 1 than those using it in tutor mode (t = 2.17, f = 38.24, p = 0.036) (Table 2).

**Table 2:** Relationship of tutor or timed mode with exam performance USMLE: United States Medical Licensing Examination; COMLEX-USA: Comprehensive Osteopathic Medical Licensing Examination of the United States; COMQUEST and COMBANK (question banks for COMLEX-USA)

	Timed	Tutor	Two Sample T Test
UWorld Mode related to USMLE	mean= 236.9	mean=230.4	t = 1.05 df = 14.26 p=0.310
UWorld Mode related to COMLEX-USA	mean=638.4	mean=594.4	t = 2.04 df = 34.67 p = 0.049
USMLE Rx Mode related to USMLE	mean=241.8	mean=254.8	t = -1.90 df = 9.35 p = 0.089
USMLE Rx Mode related to COMLEX-USA	mean=660.1	mean=639.9	t = 0.52, df = 14.65 p = 0.611
Kaplan USMLE Mode related to USMLE	mean=238.4	mean=234.1	t = 0.68 df = 15.69 p-value = 0.508
Kaplan USMLE Mode related to COMLEX-USA	mean=644.9	mean=589.1	t = 2.17 f = 38.24 p = 0.036
Truelearn COMBANK Mode related to USMLE	mean=239.3	mean=234.1	t = 0.91 df = 23.3 p = 0.374
Truelearn COMBANK Mode related to COMLEX-USA	mean=642.0	mean=589.0	t = 2.36 df = 44 p = 0.023
COMQUEST Mode related to USMLE	mean=231.5	mean=243.0	t = -0.99 df = 5.99 p-value = 0.360
COMQUEST Mode related to COMLEX-USA	mean=612.3	mean=582.0	t = 0.93 df = 13.57 p = 0.370
Kaplan COMLEX Mode related to USMLE	mean=2389.4	mean=233.0	t = 0.86 df = 9.67 p = 0.410
Kaplan COMLEX Mode related to COMLEX-USA	mean=630.4	mean=578.8	t = 1.74 df = 20.88 p-value = 0.096

Practice examinations

NBME CBSSA practice tests were consistently positively correlated with performance outcomes of USMLE Step 1 (r = 0.53-0.80) and COMLEX-USA Level 1 (r = 0.55-0.73). Students’ highest performing NBME CBSSA was correlated with both the USMLE Step 1 (r = 0.82) and COMLEX-USA Level 1 scores (r = 0.74). Students’ performance on a COMSAE taken nearest to their USMLE Step 1 and COMLEX-USA Level 1 examination was positively correlated with the USMLE Step 1 (r = 0.41) and COMLEX-USA Level 1 score (r = 0.61) (Table 3).

**Table 3:** Correlation of practice exams with exam performance USMLE: United States Medical Licensing Examination; COMLEX-USA: Comprehensive Osteopathic Medical Licensing Examination of the United States; CBSSA: Comprehensive Basic Science Self Assessment; NBOME: National Board of Osteopathic Medical Examiners; COMSAE: Comprehensive Osteopathic Self-Assessment Examinations

	USMLE Step 1 Performance	COMLEX-USA Level 1 Performance	df
NBME CBSSA Practice Test			
Form 13	0.6737566	0.6958199	17
Form 15	0.525186	0.5552731	26
Form 16	0.6768368	0.7232968	24
Form 17	0.7363386	0.6113651	22
Form 18	0.80261	0.7306376	20
Form 19	0.7982869	0.687549	21
Highest Performing Form	0.8233617	0.7358066	40
NBOME COMSAE Practice Test			
COMSAE Form A (taken in December)	0.4695146	0.2513266	49(USMLE) 84
COMSAE Form E (taken in April)	0.4049869	0.6063537	32(USMLE) 61

Video resources

Prior to adjusting for multiple comparisons, Sketchy Micro (marketed by Sketchy Group, LLC, California, US) was the only resource whose total completion was related to higher scores on the COMLEX-USA (t = -2.17, p = 0.04). Total completion of video resources was not related to performance outcomes on the USMLE Step 1 (Table 4).

**Table 4:** Relationship of video resource use with exam performance USMLE: United States Medical Licensing Examination; COMLEX-USA: Comprehensive Osteopathic Medical Licensing Examination of the United States

	Not Completed	Completed	Two-Sample T-Test
Sketchy Micro related to USMLE	mean= 226	mean=234.2	t=-1.62 df=11.22 p=0.131
Sketchy Micro related to COMLEX-USA	mean=565.1	mean=607.8	t=2.17 df=32.03 p=0.038
Sketchy Pharm related to USMLE	mean=229.6	mean=236.8	t=-92 df=49.99 p=0.061
Sketchy Pharm related to COMLEX-USA	mean=587.1	mean=613.1	t=-1.63 df=94.56 p=0.107
Pathoma related to USMLE	mean=242	mean=231.9	t=2.15 df=7.30 p=0.067
Pathoma related to COMLEX-USA	mean=621.9	mean=595.2	t=0.92 df=14.16 p=0.372
Doctors in Training related to USMLE	mean=233.9	mean=220.8	t=2.45 df=3.96 p=0.070
Doctors in Training related to COMLEX-USA	mean=600.8	mean=572.4	t=1.15 df=8.93 p=0.281
Kaplan related to USMLE	mean=232.7	mean=234.6	t=-0.27 df=7.17 p=0.79
Kaplan related to COMLEX-USA	mean=594.6	mean=639.1	t=-1.35 df=9.08 p=0.207
USMLE Express related to USMLE	mean=232.9	mean=232.8	t=0.02 df=6.204 p=0.982
USMLE Express related to COMLEX-USA	mean=599.5	mean=591.9	t=0.33 df=15.17 p=0.745

## Discussion

National medical licensing board examination performance is crucial to both student success in attaining post-graduate residency training positions and in medical school accreditation. In consideration of reports that licensing board examination performance is a prime factor in selecting applicants for residency interviews [4], and the importance of examination pass rates in the accreditation of COCA-accredited medical schools [12], improving performance on these high-stakes examinations is in the interest of both students and their respective institutions. There is, presently, a paucity of published data demonstrating evidence of modifiable factors that bolster examination performance. Rather, much of the literature centers on the topic of admission criteria such as the Medical College Admission Test (MCAT) score and the undergraduate or post-baccalaureate grade-point average (GPA) [13]. There is also limited data available to the public analyzing the relationship between performance on practice examinations, such as the NBME CBSSA and NBOME COMSAE, and the eventual outcomes of the correlated high-stakes licensing examinations.

US-MD students and international medical graduates (IMGs) are required to take USMLE Step 1 while all osteopathic medical students are required to take COMLEX-USA Level 1, with the option to also take USMLE Step 1 in order to apply for medical licensure in the United States. Certain medical residency programs require that USMLE Step 1 and occasionally USMLE Step 2 CK be taken by osteopathic medical student applicants, while others will accept COMLEX-USA Level 1 and 2CE scores alone. This had led to an interest in the correlation between scores on the two examinations. A previous small-scale study determined the Pearson correlation coefficient between COMLEX-USA Level 1 and USMLE Step 1 scores to be 0.83 (p<0.001) [14]. Our study showed a similar correlation of 0.78 (p < 0.001), supporting the hypothesis that a strong positive correlation exists between COMLEX-USA Level 1 and USMLE Step 1 examination performance.

Several third-party companies offer practice questions for medical student board examination studying. The present study assessed which question banks students used, the number of questions completed, and the outcome of their performance on both USMLE Step 1 and COMLEX-USA Level 1. Consistent with our expectations, it was found that the number of practice questions students completed correlated positively with scores on both USMLE Step 1 and COMLEX-USA Level 1. Additionally, these results demonstrated that students who used question banks in timed mode performed better than students who completed these question banks in tutor mode or timed-tutor mode. It is worth noting that in this study, questions banks marketed toward preparation for USMLE Step 1 had a modestly stronger correlation with COMLEX-USA Level 1 scores than with USMLE Step 1 scores (r = 0.42 and r = 0.38, respectively). Interestingly, the data collected in this study demonstrated a negative correlation between the number of questions completed in banks marketed toward preparation for the COMLEX-USA Level 1 (r = -0.22) and COMLEX-USA Level 1 scores. This finding was particularly noteworthy given that some products, such as Truelearn COMBANK, advertise the applicability of its product to COMLEX-USA Level 1 in much of their marketing materials and student testimonials [15-16]. Osteopathic medical students who only sit for COMLEX-USA Level 1 may select these resources over those marketed for preparation for USMLE Step 1 as a result of such marketing statements and testimonials.

Practice examinations and self-assessments designed by the NBME and NBOME are tools used by medical schools and medical students in order to assess preparedness for the licensing examinations. While this study was not designed to assess the differences between NBME CBSSA and NBOME COMSAE, the strength of the correlation between scores on NBME CBSSA and the performance outcomes on COMLEX-USA Level 1 suggests that further research is needed in order to identify those self-assessments that will most reliably predict preparedness for these licensing examinations. While the COMSAE is described as a self-assessment for osteopathic medical students, the present data suggest that osteopathic medical students may not be best served in assessing their future examination performance as a direct function of their COMSAE scores.

Sketchy Micro, a microbiology review resource, was the only video resource that was related to an increase in score on COMLEX-USA Level 1. The completion of video resources did not appear to be related to the USMLE Step 1 score. Despite this finding, the present study may not have been adequately powered to reliably observe the relationship between the use of video resources and USMLE Step 1 and COMLEX-USA Level 1 performance.

This study was limited by being a convenience sample rather than a randomized trial of different resources. Due to the study population and the subject matter, it would be difficult to design such a trial where students would be encouraged to complete one resource and avoid others. Additionally, the study was limited by being self-reports of exam scores and exam preparation rather than information gathered through a data pull. This was done specifically to ensure the consent of participants in what data was and was not available, however, it remains a limitation of the above work.

## Conclusions

This study demonstrates that resources written for the USMLE Step 1 exam are effective at preparing students for the COMLEX-USA Level 1 exam, and that performance on USMLE Step 1 is highly correlated with COMLEX- USA Level 1. This study also demonstrates the need for both medical schools and students to evaluate the resources they purchase for board examination preparation, especially those that claim to be designed for COMLEX-USA Level 1. Additionally, a collaboration between LCME- and COCA-accredited medical schools and longitudinal studies is needed in order to evaluate the resources and study methods used by medical students to optimize their performance on these examinations.

## Figures and Tables

**Figure 3:**
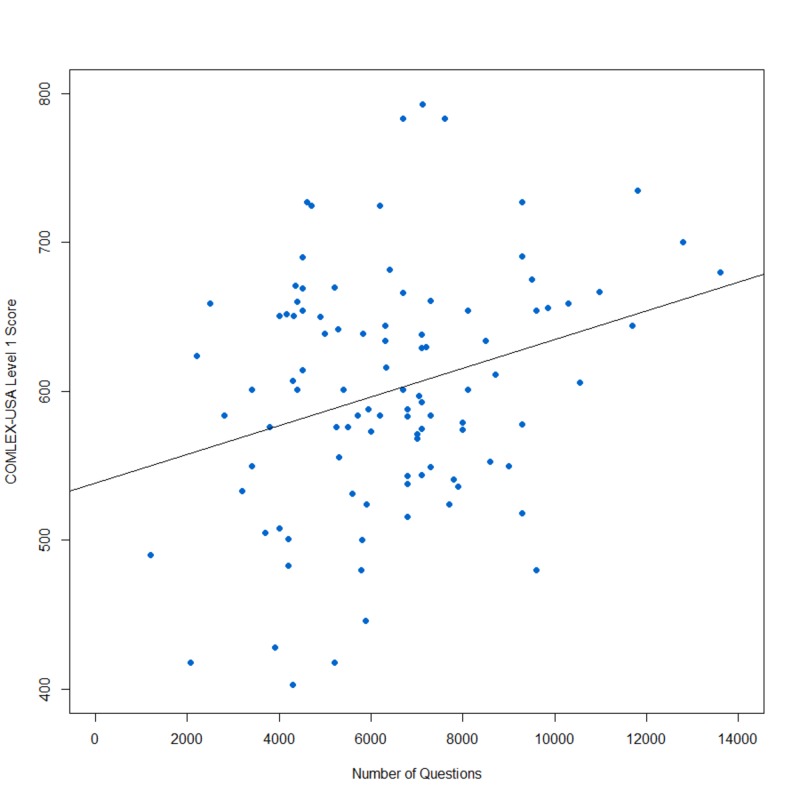
Regression of total questions performed with COMLEX-USA Score COMLEX-USA: Comprehensive Osteopathic Medical Licensing Examination of the United States

**Figure 4:**
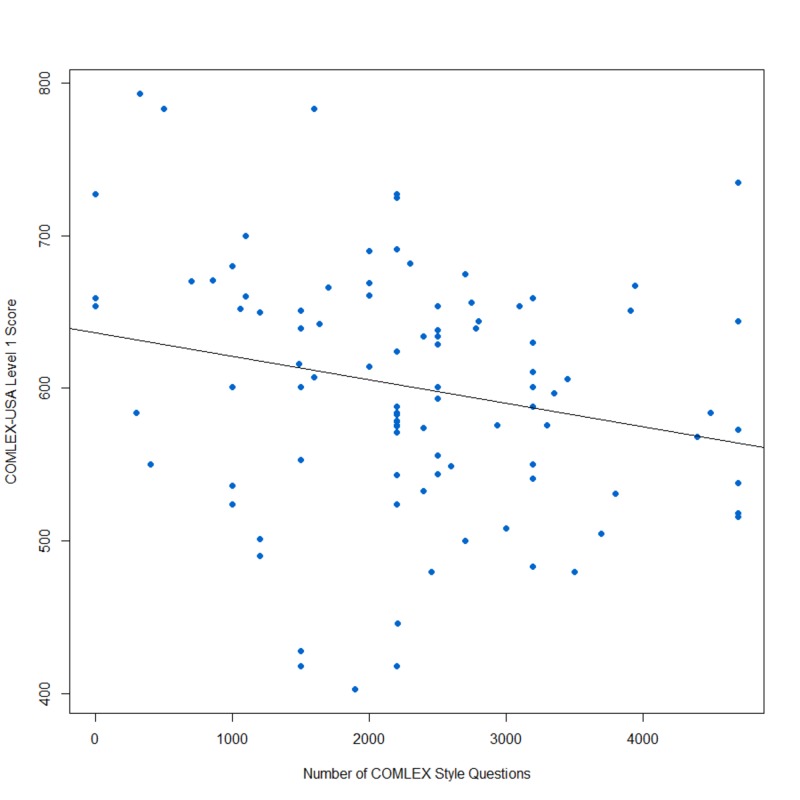
Regression of COMLEX Style Questions performed with COMLEX-USA Level 1 Score COMLEX-USA: Comprehensive Osteopathic Medical Licensing Examination of the United States

**Figure 5:**
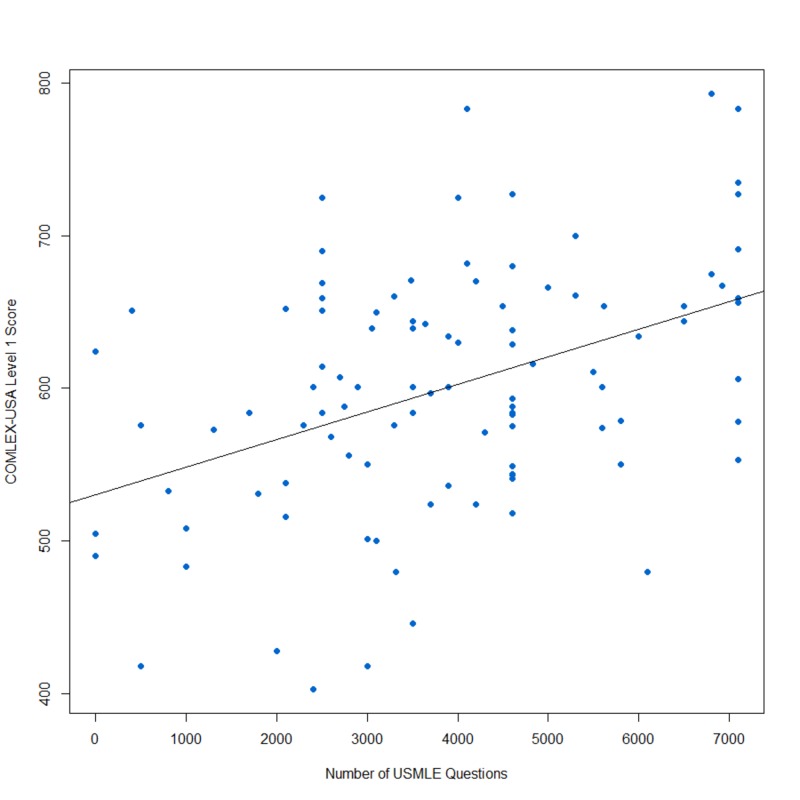
Regression of USMLE Style Questions Performed with the COMLEX-USA Level 1 Score USMLE: United States Medical Licensing Examination; COMLEX-USA: Comprehensive Osteopathic Medical Licensing Examination of the United States

**Figure 6:**
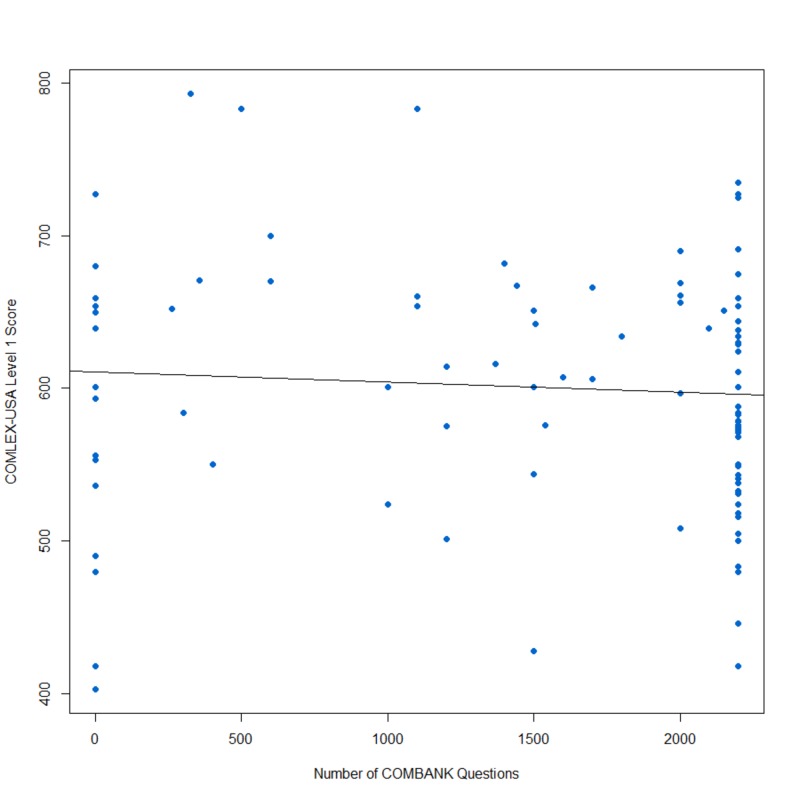
Regression of COMBANK Questions performed with COMLEX-USA Level 1 Score COMLEX-USA: Comprehensive Osteopathic Medical Licensing Examination of the United States; COMBANK (question bank for COMLEX-USA)

**Figure 7:**
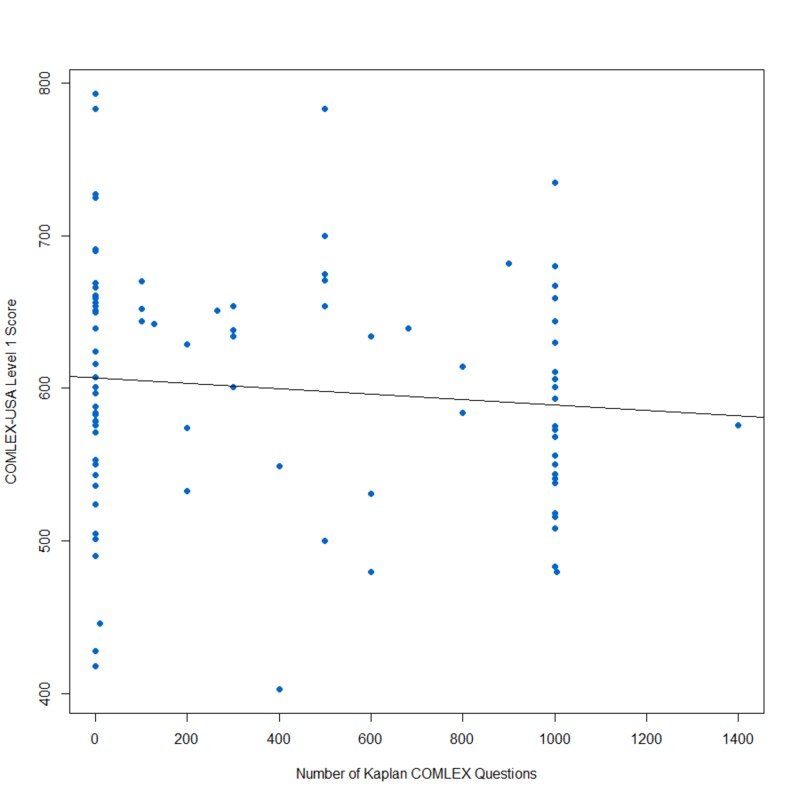
Regression of Kaplan COMLEX Questions performed with COMLEX-USA Level 1 Score COMLEX-USA: Comprehensive Osteopathic Medical Licensing Examination of the United States

**Figure 8:**
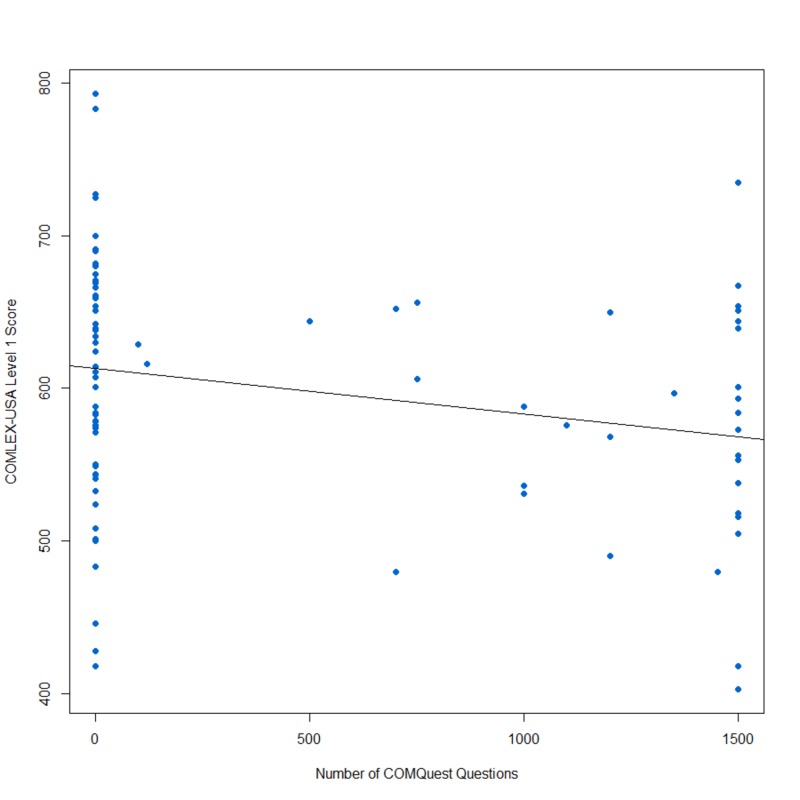
Regression of COMQuest Questions performed with COMLEX-USA Level 1 Score COMLEX-USA: Comprehensive Osteopathic Medical Licensing Examination of the United States; COMQUEST (question bank for COMLEX-USA)

**Figure 9:**
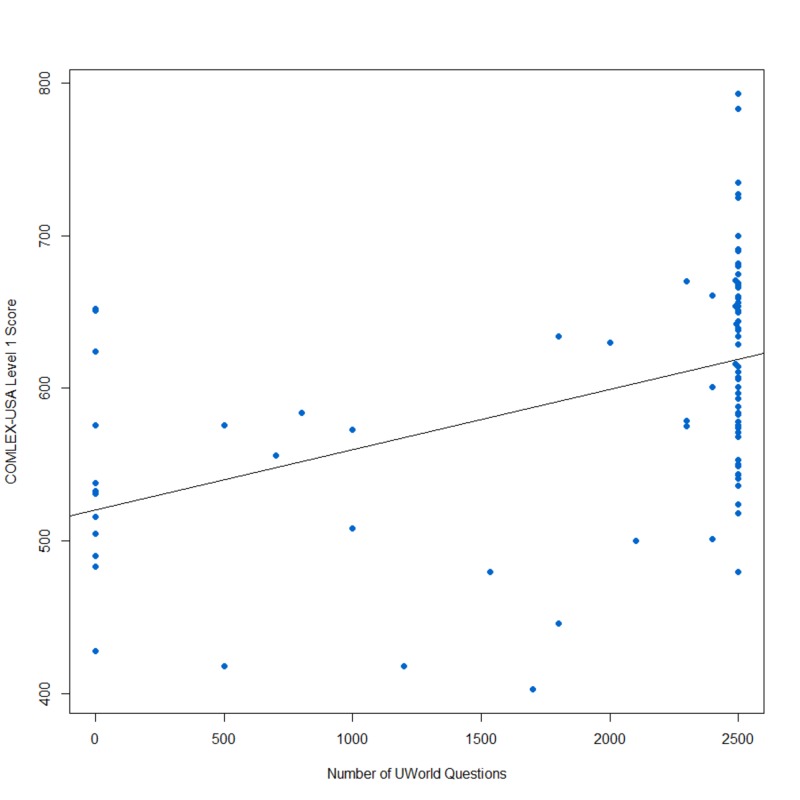
Regression of UWorld Questions performed with COMLEX-USA Level 1 Score COMLEX-USA: Comprehensive Osteopathic Medical Licensing Examination of the United States

**Figure 10:**
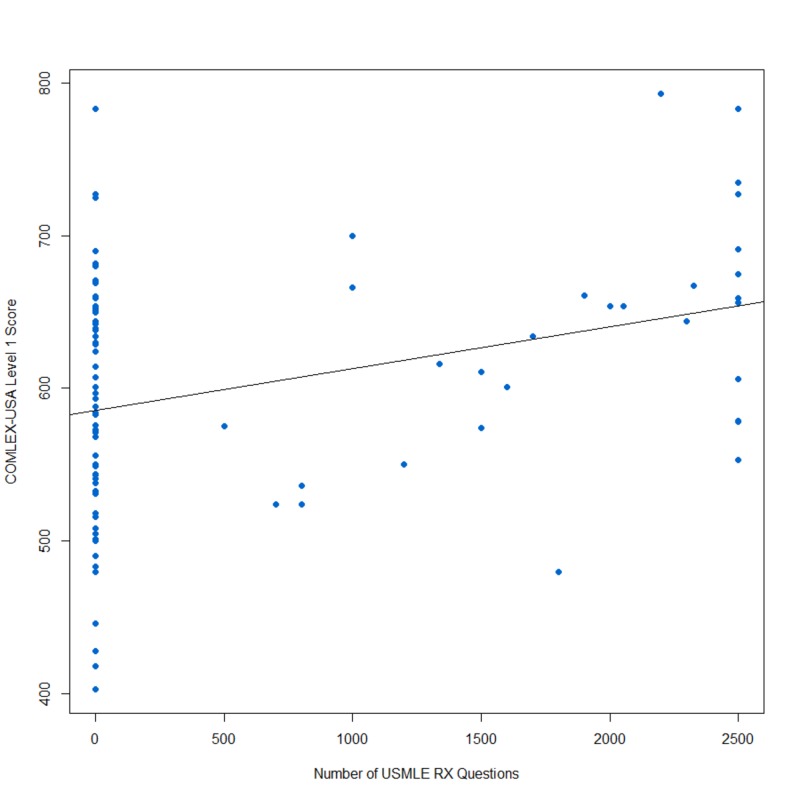
Regression of USMLE-Rx Questions performed with COMLEX-USA Level 1 Score USMLE: United States Medical Licensing Examination; COMLEX-USA: Comprehensive Osteopathic Medical Licensing Examination of the United States

**Figure 11:**
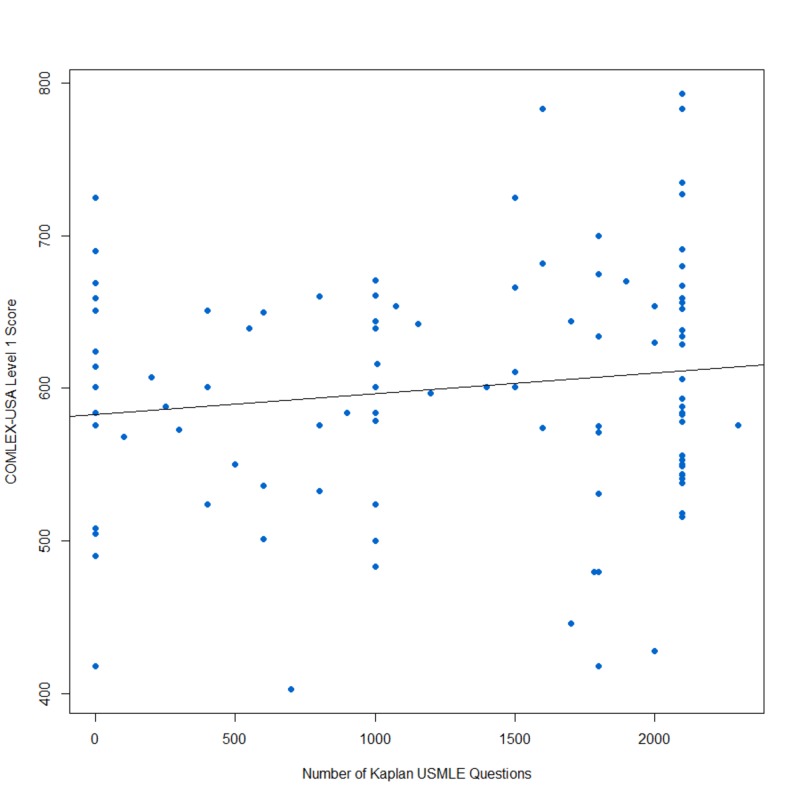
Regression of Kaplan USMLE Questions performed with COMLEX-USA Level 1 Score USMLE: United States Medical Licensing Examination; COMLEX-USA: Comprehensive Osteopathic Medical Licensing Examination of the United States

**Figure 12:**
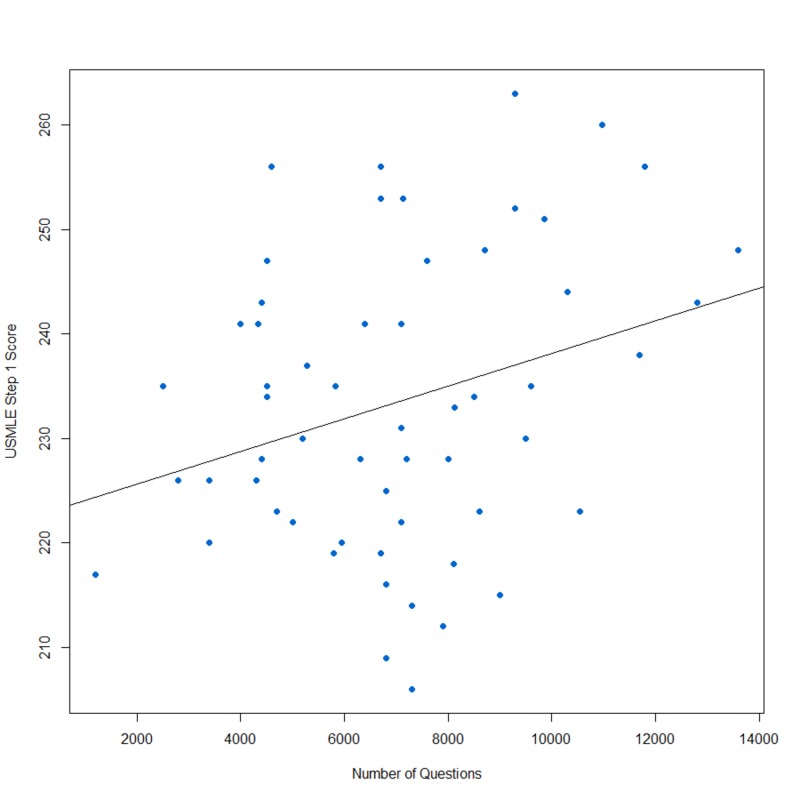
Regression of Total Questions performed with USMLE Step 1 Score USMLE: United States Medical Licensing Examination

**Figure 13:**
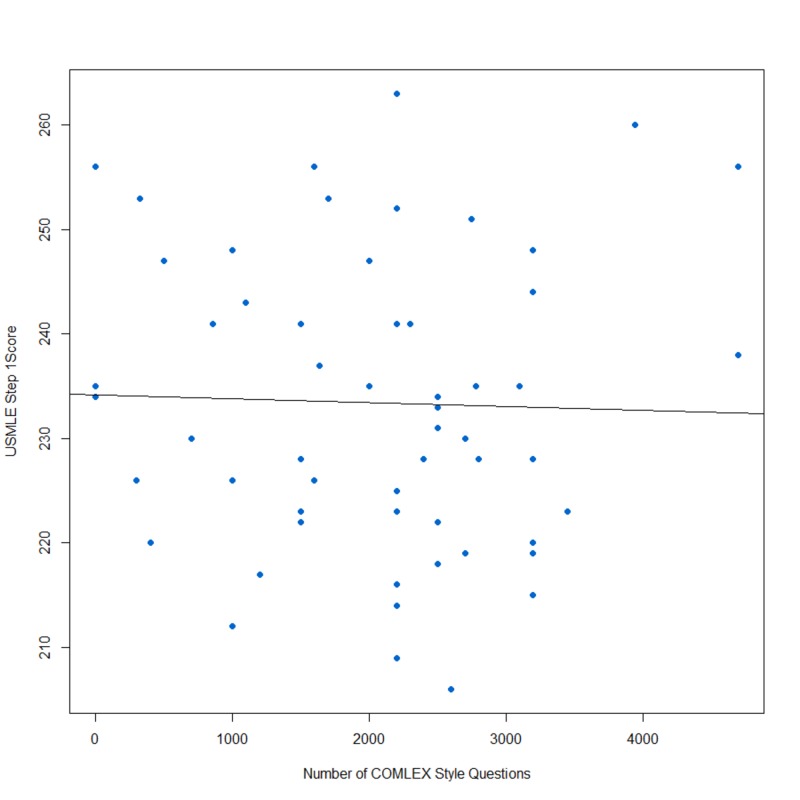
Regression of COMLEX Style performed with USMLE Step 1 Score USMLE: United States Medical Licensing Examination; COMLEX-USA: Comprehensive Osteopathic Medical Licensing Examination of the United States

**Figure 14:**
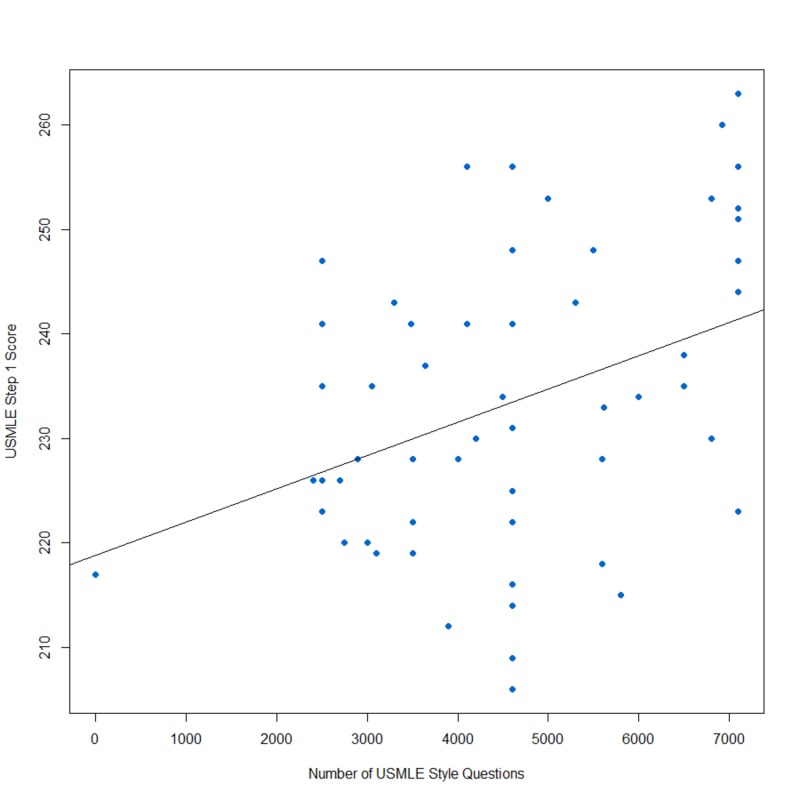
Regression of USMLE Style performed with USMLE Step 1 Score USMLE: United States Medical Licensing Examination

**Figure 15:**
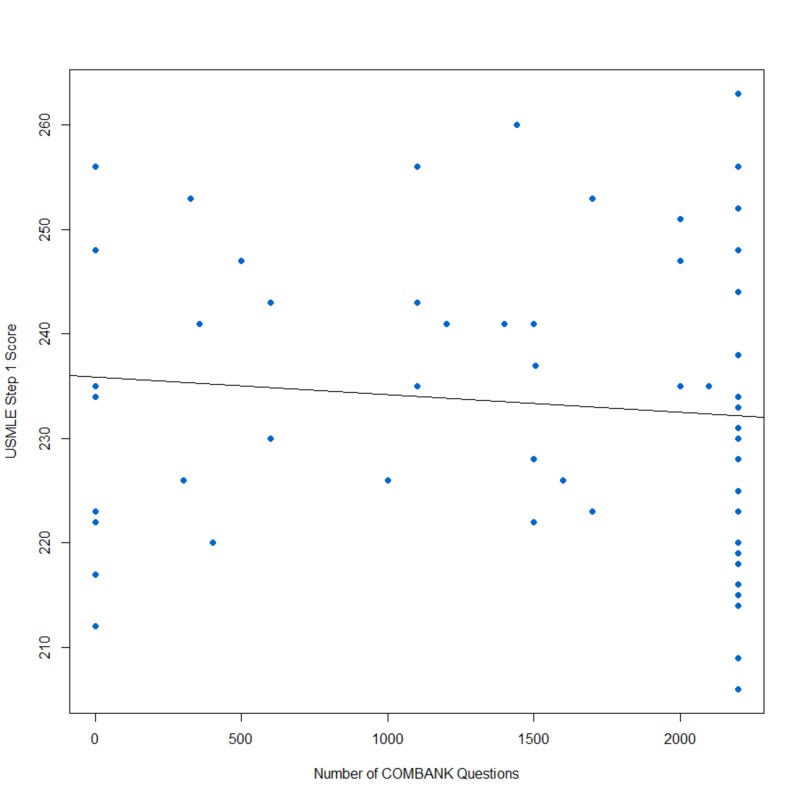
Regression of COMBANK questions performed with USMLE Step 1 Score USMLE: United States Medical Licensing Examination; COMBANK (question bank for COMLEX-USA)

**Figure 16:**
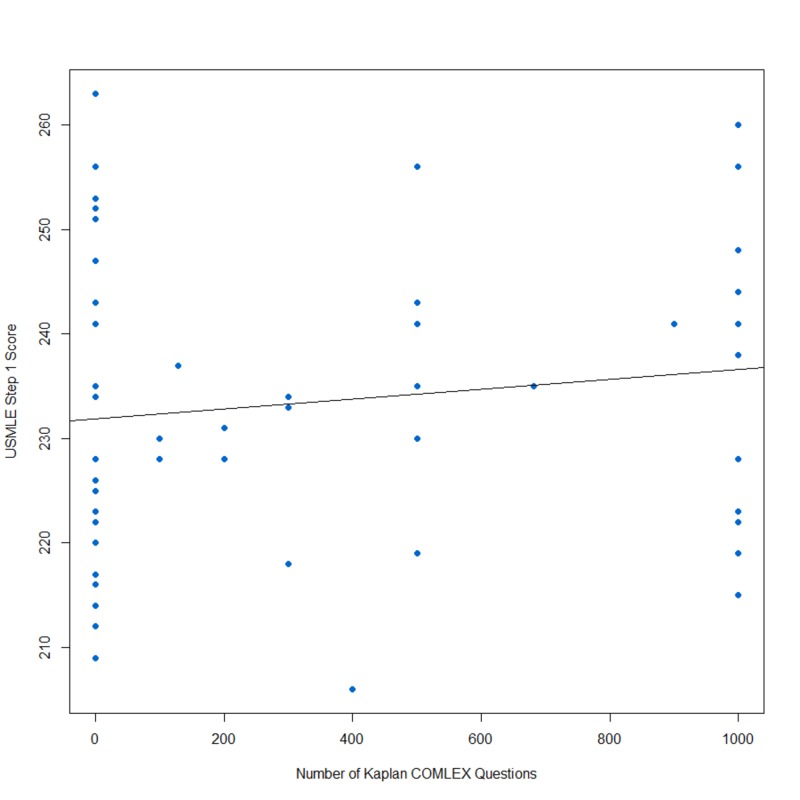
Regression of Kaplan COMLEX questions performed with USMLE Step 1 Score United States Medical Licensing Examination; COMLEX (question bank for COMLEX-USA)

**Figure 17:**
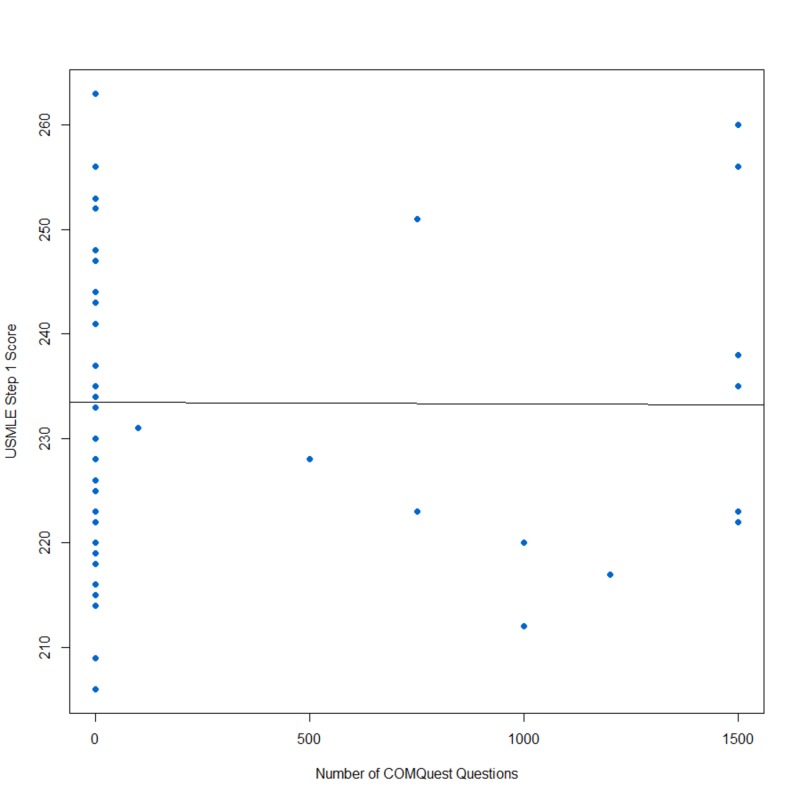
Regression of COMQuest questions performed with USMLE Step 1 Score USMLE: United States Medical Licensing Examination; COMQuest (question bank for COMLEX-USA)

**Figure 18:**
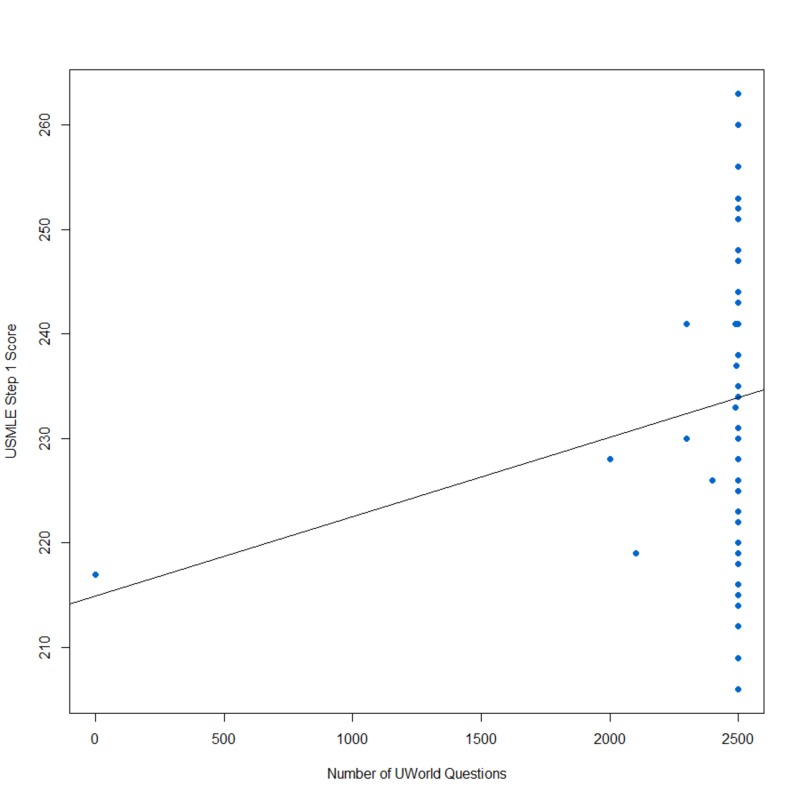
Regression of UWorld questions performed with USMLE Step 1 Score USMLE: United States Medical Licensing Examination

**Figure 19:**
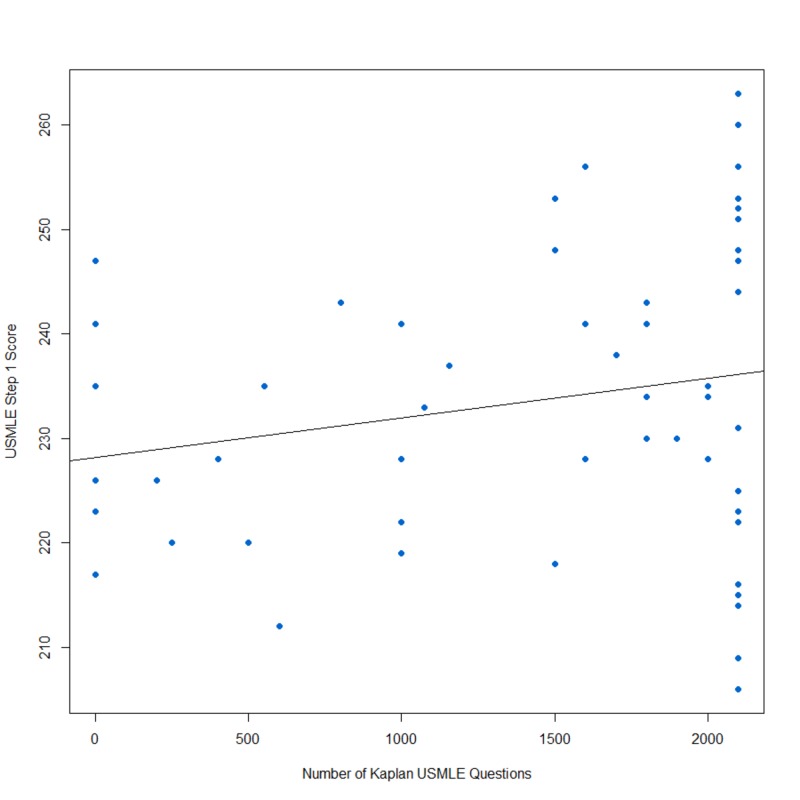
Regression of Kaplan USMLE questions performed with USMLE Step 1 Score USMLE: United States Medical Licensing Examination

## Appendices

This data is included and presented in the question bank subsection of the Results section, specifically as part of Table 1. The following figures are presented for additional reference.
